# Supplementary material for: GC-FID-MS Based Metabolomics to Access Plum Brandy Quality
Source: Molecules. 2021 Mar 5;26(5):1391. doi: 10.3390/molecules26051391 (PMC7961760; doi:10.3390/molecules26051391)
Supplement: Supplementary file 1 [file molecules-26-01391-s001.pdf]

Table S1. The plum mash experiments of Požegača, Crvena ranka and Trnovača cultivars depending on the added of enzyme, yeast (0-not added. 1-added) and pH values.

Experiment[1]	Yeast	Lallzyme BETA™	CUVÉE BLANC™	pH of plum mash
I	0	0	0	3.5
II	0	1	0	3.5
III	0	0	1	3.5
IV	0	0	0	3
V	0	1	0	3
VI	0	0	1	3
VII	1	0	0	3.5
VIII	1	1	0	3.5
IX	1	0	1	3.5
X	1	0	0	3
XI	1	1	0	3
XII	1	0	1	3

[1]The 12 experiments were performed for each cultivar, and in triplicate.

Table S2. The main volatile compounds from plum brandy (GC-FID)

No.	RT(min)	Compound
1	1.124	Acetaldehide
2	1.738	Etil acetat
3	1.825	Metanol
4	3.686	*n*-Propanol
5	5.483	*i*-Butanol
6	7.725	*n*-Butanol
7	11.329	*iso*-amil alkohol
8	17.795	*n*-Heksanol

Table S3. Aroma compounds from plum brandy (GC-FID/MSA). Part 1

No.	Rt (min)	RI	Compound
1	3.374	804	Ethyl butyrate
2	3.563	815	Ethyl lactate
3	3.849	837	Furfural
4	4.010	844	2-methylbutanoic acid
5	4.133	845	2-methylbutanoic acid, ethyl ester
6	4.188	855	(Z)-3-hexen-1-ol
7	4.388	866	1-hexanol
8	4.534	875	3-methyl-1-butanol, acetate
9	4.632	876	2-methyl-1-butanol, acetate
10	5.072	905	1,1-diethoxybutane
11	6.684	964	3,3-diethoxy-2-butanone
12	6.808	968	1-Heptanol
13	6.972	971	1-(1-ethoxyethoxy)pentane
14	7.634	999	1,1-diethoxypentane
15	7.666	1001	Hexanoic acid, ethyl ester
16	7.850	1009	Hexanoic acid
17	8.135	1014	3-Z-Hexenyl acetate
18	8.166	1016	Hexyl acetate
19	8.856	1040	Benzyl alcohol
20	9.736	1059	Ethyl 2-hydroxy-4-methylpentanoate
21	10.163	1070	Isoamyl lactate
22	10.263	1074	1-Octanol
23	10.561	1080	1,3-triethoxypropane
24	10.964	1091	E-linalool oxide II (furanoid)
25	11.023	1095	1,1-diethoxyhexane
26	11.408	1102	Linalol
27	11.580	1105	Nonanal
28	12.106	1117	Phenylethyl Alcohol
29	12.408	1126	Octanoic acid, methyl ester
30	13.681	1153	p-vinylanisole

Table S3. Aroma compounds from plum brandy (GC-FID/MSA). Part 2

No.	Rt (min)	RI	Compound
31	14.086	1162	Benzyl acetate
32	14.398	1170	Benzoic acid, ethyl ester
33	14.845	1176	Octanoic acid
34	14.864	1188	Diethyl succinate
35	15.207	1190	1,1-Diethoxyheptane
36	15.276	1191	α-Terpineol
37	15.428	1197	Octanoic acid, ethyl ester
38	16.567	1218	Cyclocitral <beta>
39	17.155	1234	4,4,5-trimethyl-2-pentil-1,3-dioxolane
40	17.61	1247	Ethyl phenyleacetate
41	18.143	1258	Acetic acid, 2-phenylethyl ester
42	18.737	1271	Ethyl salicylate
43	19.510	1288	1,1 diethoxyoctane
44	19.747	1293	Octanoic acid, propyl ester
45	19.874	1297	Nonanoic acid, ethyl ester
46	20.630	1313	Acetic acid, nonyl ester
47	20.749	1317	Guaiacol <para-vinyl->
48	21.296	1325	Decanoic acid, methyl ester
49	22.648	1359	Eugenol
50	23.117	1370	3,4-Dimethoxystyrene
51	23.248	1374	Decanoic acid
52	23.620	1381	Ethyl trans-4-decenoate
53	23.819	1385	1,1-diethoxynonane
54	24.337	1397	Decanoic acid, ethyl ester
55	26.093	1438	2-Methylbutyl benzoate

Table S3. Aroma compounds from plum brandy (GC-FID/MSA). Part 3

No.	Rt (min)	RI	Compound
56	26.426	1448	Octanoic acid, 3-methylbuthyl ester
57	26.583	1451	Octanoic acid, 2-Methylbutyl ester
58	26.625	1455	4-methoxybenzoic acid, ethyl ester
59	27.315	1468	Ethyl cinnamate
60	27.398	1469	Decalactone <gamma>
61	28.174	1489	Jonone (E) beta
62	28.348	1491	Decanoic acid, propyl ester
63	29.621	1527	Dodecanoic acid, methyl ester
64	31.335	1568	Nerolidol <E>
65	31.913	1571	Dodecanoic acid
66	32.030	1576	Octanoic acid, ethyl ester
67	32.660	1595	Dodecanoic acid, ethyl ester
68	34.468	1646	Decanoic acid, 3-methylbutyl ester
69	34.591	1650	Decanoic acid, 2-methylbutyl ester
70	35.322	1664	Syringaldehyde
71	35.945	1680	γ-Dodecalactone
72	36.264	1690	Dodecanoic acid, propyl ester
73	37.645	1728	Ethyl tetradecadienoat
74	38.758	1757	Ethyl tetradecadienoat, isomer 2
75	40.252	1795	Tetradecanoic acid, ethyl ester
76	41.831	1844	Dodecanoic acid, 3-methylbutyl ester
77	42.132	1848	Octanoic acid, 2-phenylethyl ester
78	43.805	1898	Pentadecanoic acid, ethyl ester
79	44.893	1929	Hexadecanoic acid, methyl ester
80	46.474	1970	9-hexadecenoic acid, ethyl ester

Table S3. Aroma compounds from plum brandy (GC-FID/MSA). Part 4

No.	Rt (min)	RI	Compound
81	47.260	1996	Hexadecanoic acid, ethyl ester
82	50.238	2103	Methyl linolelaidate
83	50.444	2110	Octadecenoic acid, methyl ester
84	52.556	2175	Linoleic acid, ethyl ester
85	52.738	2181	Linolenic acid, ethyl ester
86	52.864	2186	Oleic acid, ethyl ester
87	53.473	2206	Octadecanoic acid, ethyl ester
88	54.778	2257	Hexadecanoic acid, 3-methylbutyl ester
89	70.162	2840	Squalene

Table S4. Misclassification tables of the OPLS-DA models

M2: cultivars CR/PZ

	**Members**	**Correct**	**CR**	**PZ**	**No class (YPred <= 0)**
**CR**	36	100%	36	0	0
**PZ**	36	100%	0	36	0
**No class**	0		0	0	0
Total	72	100%	36	36	0
Fisher's prob.	2.3e-21				

M3: cultivars TR/PZ

	Members	Correct	TR	PZ	No class (YPred <= 0)
**TR**	36	100%	36	0	0
**PZ**	36	100%	0	36	0
**No class**	0		0	0	0
Total	72	100%	36	36	0
Fisher's prob.	2.3e-21				

M4: pH of plum mash

	Members	Correct	pH 3.0	pH 3.5	No class (YPred <= 0)
**pH 3.0**	54	100%	54	0	0
**pH 3.5**	54	100%	0	54	0
**No class**	0		0	0	0
Total	108	100%	54	54	0

Fisher's prob. 4e-32

M5: natural/selected yeast

	Members	Correct	Natural	Lalvin QA23	No class (YPred <= 0)
**Natural**	54	100%	54	0	0
**Lalvin QA23**	54	100%	0	54	0
**No class**	0		0	0	0
Total	108	100%	54	54	0

Fisher's prob. 4e-32

M6: with/without Beta

	Members	Correct	Without Beta	With Beta	No class (YPred <= 0)
**Without Beta**	36	100%	36	0	0
**With Beta**	36	100%	0	36	0
**No class**	0		0	0	0
Total	72	100%	36	36	0
Fisher's prob.	2.3e-21				

M7: with/without Cuvee

	Members	Correct	Without Cuvee	With Cuvee	No class (YPred <= 0)
**Without Cuvee**	36	63.89%	23	13	0
**With Cuvee**	36	61.11%	14	22	0
**No class**	0		0	0	0
Total	72	62.5%	37	35	0
Fisher's prob.	0.029				
